# Single amino acid substitutions in hydrophobic cores at a head-coiled coil junction region of cohesin facilitate its release of DNA during anaphase

**DOI:** 10.1098/rsob.210275

**Published:** 2022-04-27

**Authors:** Xingya Xu, Ryuta Kanai, Li Wang, Mitsuhiro Yanagida

**Affiliations:** ^1^ G0 Cell Unit, Okinawa Institute of Science and Technology Graduate University, Onna-son, Okinawa 904-0495, Japan; ^2^ Institute of Quantitative Biosciences, The University of Tokyo, 113-0032 Tokyo, Japan

**Keywords:** saturation mutagenesis, suppressor screen, cohesin, hydrophobic core, coiled coils

## Abstract

Cohesin holds sister chromatids together and is cleaved by separase/Cut1 to release DNA during the transition from mitotic metaphase to anaphase. The cohesin complex consists of heterodimeric structural maintenance of chromosomes (SMC) subunits (Psm1 and Psm3), which possess a head and a hinge, separated by long coiled coils. Non-SMC subunits (Rad21, Psc3 and Mis4) bind to the SMC heads. Kleisin/Rad21's N-terminal domain (Rad21-NTD) interacts with Psm3's head-coiled coil junction (Psm3-HCJ). Spontaneous mutations that rescued the cleavage defects in temperature-sensitive (ts) separase mutants were identified in the interaction interface, but the underlying mechanism is yet to be understood. Here, we performed site-directed random mutagenesis to introduce single amino acid substitutions in Psm3-HCJ and Rad21-NTD, and then identified 300 mutations that rescued the cohesin-releasing defects in a separase ts mutant. Mutational analysis indicated that the amino acids involved in hydrophobic cores (which may be in close contact) in Psm3-HCJ and Rad21-NTD are hotspots, since 80 mutations (approx. 27%) were mapped in these locations. Properties of these substitutions indicate that they destabilize the interaction between the Psm3 head and Rad21-NTD. Thus, they may facilitate sister chromatid separation in a cleavage-independent way through cohesin structural re-arrangement.

## Graphical abstract

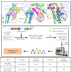

## 1. Introduction

During the cell cycle, chromosomal DNA replicates in S-phase to form two identical sets called sister chromatids (SCs). They are held together by a chromatin-associated protein complex, called cohesin, until the onset of anaphase [1,2]. During anaphase of mitosis, the activated protease, separase Cut1, cleaves the cohesin subunit Rad21/kleisin so that the SCs can separate and move towards opposite spindle poles [3–7]. Finally, the two daughter cells inherit identical genetic material. Unequal chromosome segregation may result in cell death [8] or diseases. Cornelia de Lange syndrome, a developmental disorder, has been linked to dysfunction of cohesin [9,10] or of HDAC8, the lysine deacetylase of the cohesin SMC3 subunit [11].

Cohesin is a heteropentameric protein complex that contains five essential subunits: Psm1/SMC1, Psm3/SMC3, Rad21/Kleisin, Mis4/SCC2/NIPBL and Psc3/STAG1-3 [12]. Psm1/SMC1 and Psm3/SMC3 are heterodimeric structural maintenance of chromosomes (SMC) subunits that possess a head domain (containing ATPase) and a hinge domain, which are connected by long coiled coils [13]. The Psm1 hinge and Psm3 hinge form a doughnut-shaped structure with two dimerization interfaces that feature a conserved arrangement of glycine residues (a GX6GX3GG sequence motif) [14,15]. Together, the Psm1 and Psm3 heads form the globular head domain that shares significant similarity with ATP-binding cassette (ABC) transporters [16]. Cohesin's ATPase domain possesses two ATPase active sites and each one contains the Walker A and Walker B consensus sequences found in most ATPases [17]. Cohesin's head and hinge domains are separated approximately 50 nm by long SMC coiled coils [14]. The three non-SMC subunits (Rad21, Psc3, and Mis4) bind to the SMC heads or the coiled coils emerging from the heads [18,19]. The Rad21/kleisin N-terminal domain (Rad21-NTD), which contains a helix–turn–helix (HTH) motif, interacts with Psm3's coiled coils close to the head domain [20,21]. Rad21/kleisin's C-terminal domain interacts with Psm1's head domain [22]. Two Cut1/separase cleavage sites (R179 and R231) occur in Rad21, and cohesin is released from chromosomal DNA during anaphase when activated Cut1/separase (after degradation of its inhibitor Cut2/securin by the anaphase promoting complex) cleaves Rad21/kleisin at these two sites [5,6,12,23–27]. Mis4/NIPBL, which has a hook-shaped structure, has DNA-binding activity and serves as the cohesin loader [28–30].

Suppressor screening in combination with an efficient and cost-effective suppressor mutation identification method using next-generation sequencing have proven useful in functional dissection of cohesin organization and dynamics [31–33]. Multiple suppressor mutations that rescue the defective Cut1/separase have been found at interfaces among cohesin subunits in either the head or hinge domain. Some of them were mapped in Rad21-NTD. In addition, several mutations were identified in Psm3's head-coiled coil junction (Psm3-HCJ: Glu95-Tyr168), which connects the head domain with the coiled coil. Therefore, Rad21-NTD and Psm3-HCJ may collaborate to enable the stable association of cohesin with chromosomal DNA.

## 2. Results

### 2.1. Saturation mutagenesis followed by suppressor screening in Psm3-HCJ and Rad21-NTD

**Figure 1.**
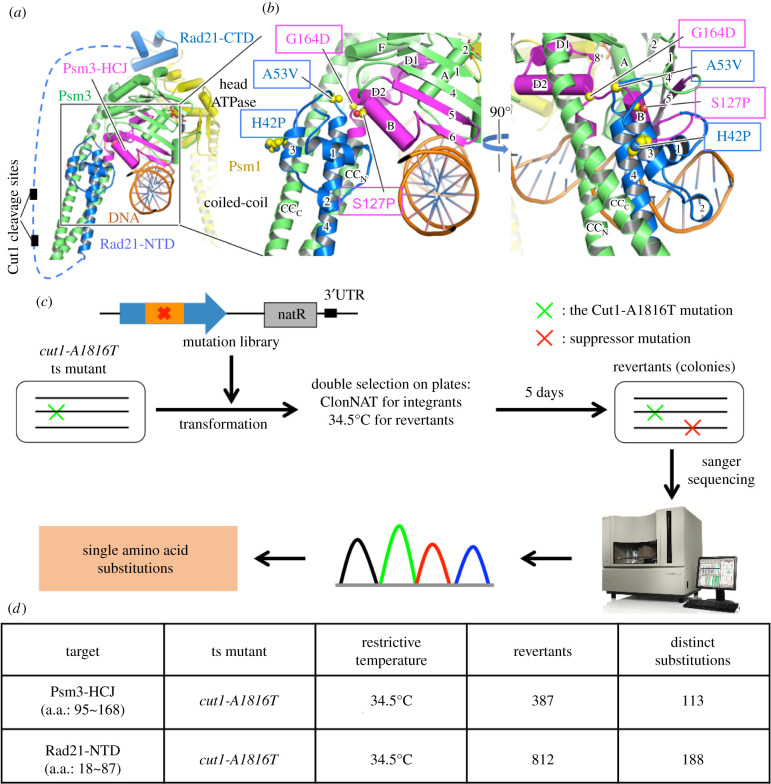
Procedures for targeted mutagenesis in the Psm3-HCJ and Rad21-NTD domains. (*a*) Structural views of cohesin Psm3, Psm1 and Rad21 (PDB code 6YUF). Yellow, green, blue and orange are used for Psm1, Psm3, Rad21 and DNA, respectively, and Psm3-HCJ is coloured in magenta. All helices are represented as cylinders, except for helices in the coiled coils. The unstructured middle region of Rad21 is presented as a dashed blue line and the two Cut1/separase cleavage sites in Rad21 (R179 and R231) are indicated by two small black rectangles. (*b*) Locations of the four mutation sites identified in Psm3-HCJ (S127P and G164D) and Rad21-NTD (H42P and A53V) as spontaneous suppressors of *cut1/cut2* ts mutants. The C*α* atoms and side chains of suppressor sites are represented as spheres. (*c*) Experimental strategy to identify single amino acid substitutions in Psm3-HCJ or Rad21-NTD that prevent lethality of the *cut1-A1816T* ts mutant at the restrictive temperature. (*d*) A summary of conditions used for genetic screens performed and results obtained. Responsible suppressor mutations in revertants were identified by Sanger sequencing of the targeted regions. The 113 substitutions identified in Psm3-HCJ and the 188 substitutions identified in Rad21-NTD are presented in figure 2*b,d*, respectively.

In our previous study, spontaneous suppressor screening for temperature-sensitive (ts) mutants of separase/Cut1 or its chaperone, securin/Cut2, in which cohesin cleavage is defective at the restrictive temperature identified multiple single amino acid substitutions with next-generation sequencing of revertants [31]. Many of them were mapped to subunits of the cohesin complex, and mostly at interfaces between cohesin subunits, according to the recent cryogenic electron microscopy (cryo-EM) structure of the cohesin complex [19]. The Rad21 N-terminal domain (Rad21-NTD) interacts with the Psm3 head and coiled coil, including the junction between them (Psm3-HCJ), as described in detail below (figure 1*a,b*). Two mutations in Rad21-NTD (Rad21-H42P and Rad21-A53V) and two mutations in Psm3-HCJ (Psm3-S127P and Psm3-G164D) were identified as suppressors of *cut1/cut2* ts mutants, whereas three of them (Rad21-A53V, Psm3-S127P and Psm3-G164D) are located close to each other (figure 1*b*).

These separase/Cut1 suppressors seem not to affect Rad21 cleavage, as they are not close to the separase/Cut1 cleavage sites in Rad21 (R179 and R231) (indicated by two small black rectangles in figure 1*a*). Therefore, to understand how Psm3-HCJ and Rad21-NTD are implicated in suppression of cohesin's failure to dissociate from chromosomal DNA and how they may regulate cohesin's association with chromosomal DNA, we performed mutagenesis concentrated in these two regions.

Seventy-four consecutive amino acid residues in Psm3-HCJ from Glu95 to Tyr168 of Psm3 and 70 consecutive amino acid residues from Trp18 to Lys87 of Rad21 were selected for mutagenesis. Site-directed saturation mutagenesis was performed for each of these amino acids in Psm3-HCJ and Rad21-NTD to introduce random ‘NNN’ codons, which replace a targeted amino acid with any of the 20 amino acids (or stop codons) (electronic supplementary material, figure S1A,B). Targeted suppressor screening was then performed, by transforming the mutational DNA library (electronic supplementary material, figure S1C) into the *cut1-A1816T* ts mutant, followed by double selection of revertants, to identify single amino acid substitutions in Rad21-NTD or Psm3-HCJ that rescue the temperature sensitivity of the *cut1-A1816T* ts mutant at its restrictive temperature 34.5°C (figure 1*c*). In summary, we identified 113 single amino acid substitutions in Psm3-HCJ and 188 single amino acid substitutions in Rad21-NTD from revertants of the *cut1-A1816T* ts mutant, respectively (figure 1*d*). No premature stop codons were detected in the screens and this is consistent with the essential functions of Psm3 and Rad21 in fission yeast [12].

To estimate how close our screens are to saturation, we calculated the number of hits for each single amino acid substitution (electronic supplementary material, figure S1D). About 70% of the suppressors in Rad21-NTD and 50% of the suppressors in Psm3-HCJ were identified in at least two revertants. Although the screens may not be saturated, they should be very close to it, especially the suppressor screen in Rad21-NTD.

### 2.2. Single amino acid substitutions in Psm3-HCJ

**Figure 2.**
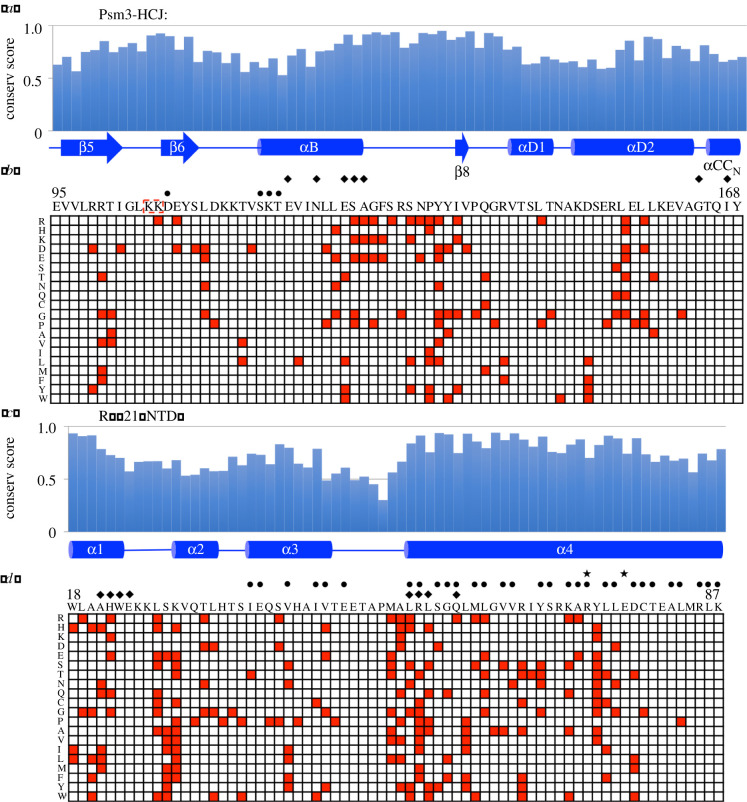
Single amino acid substitutions in Psm3-HCJ or Rad21-NTD that rescued cohesin-releasing defects in the *cut1-A1816T* ts mutant. (*a*) Relative evolutionary conservation scores (ECSs) were plotted against the amino acid sequence of Psm3-HCJ. The secondary structure of the wild-type sequence of Psm3-HCJ is depicted below the histogram. (*b*) Data matrix showing single amino acid substitutions identified in Psm3-HCJ (red squares) that rescued the temperature lethality of the *cut1-A1816T* ts mutant. Columns in the matrix depict positions along the sequence of Psm3-HCJ that are shown above the matrix, and rows indicate mutations to the 20 kinds of amino acids, shown with one-letter codes on the left of the data matrix. Amino acids that may bind DNA are indicated by ‘circles’; amino acids that may contact Rad21-NTD are indicated by ‘diamonds’. (*c*) Relative ECSs are plotted against amino acid positions in Rad21-NTD. The secondary structure of the wild-type sequence of Rad21-NTD is depicted below the histogram. (*d*) Data matrix showing all potential single amino acid substitutions in Rad21-NTD (red squares) that rescued the temperature lethality of the *cut1-A1816T* ts mutant. Amino acids that may contact Psm3-HCJ are indicated by ‘diamonds’. Amino acids that may interact with Psm3 coiled coils are indicated by ‘circles’; amino acids that are close to Mis4 are indicated by ‘stars’.

Psm3-HCJ contains four α-helices (αB, αD1, αD2 and a part of αCC_N_) and three β-strands (β5, β6, and β8). Evolutionary conservation scores for each amino acid along Psm3-HCJ, calculated from a multiple sequence alignment of homologous protein sequences [34], are shown above the secondary structure of Psm3-HCJ (figure 2*a*). One hundred and thirteen single amino acid substitutions in Psm3-HCJ that were identified as suppressors of the *cut1-A1816T* ts mutant are shown in a data matrix (figure 2*b*). Each grid represents one potential single amino acid substitution and all potential single amino acid substitutions in Psm3-HCJ are shown in the data matrix. Red grids indicate the single amino acid substitutions in Psm3-HCJ that are identified as suppressors of the *cut1-A1816T* ts mutant. Single amino acid substitutions are not evenly distributed. Those that are more vulnerable to functional alternation (those positions that have more single amino acid substitutions identified as *cut1-A1816T* suppressors) tend to be more evolutionarily conserved (figure 2*a,b*).

Psm3-HCJ contains two acetylation sites at K105 and K106 (figure 2*b*). The non-acetylatable K106R mutant is viable, but generates a cohesion defect [35]. Psm3-K106R was identified as a suppressor mutation of *cut1-A1816T* in this study, which is consistent with the hypothesis that *cut1-A1816T* suppressors in cohesin impair cohesin binding to chromatin and assist cohesin's release from chromatin [31].

### 2.3. Single amino acid substitutions in Rad21-NTD

**Figure 3.**
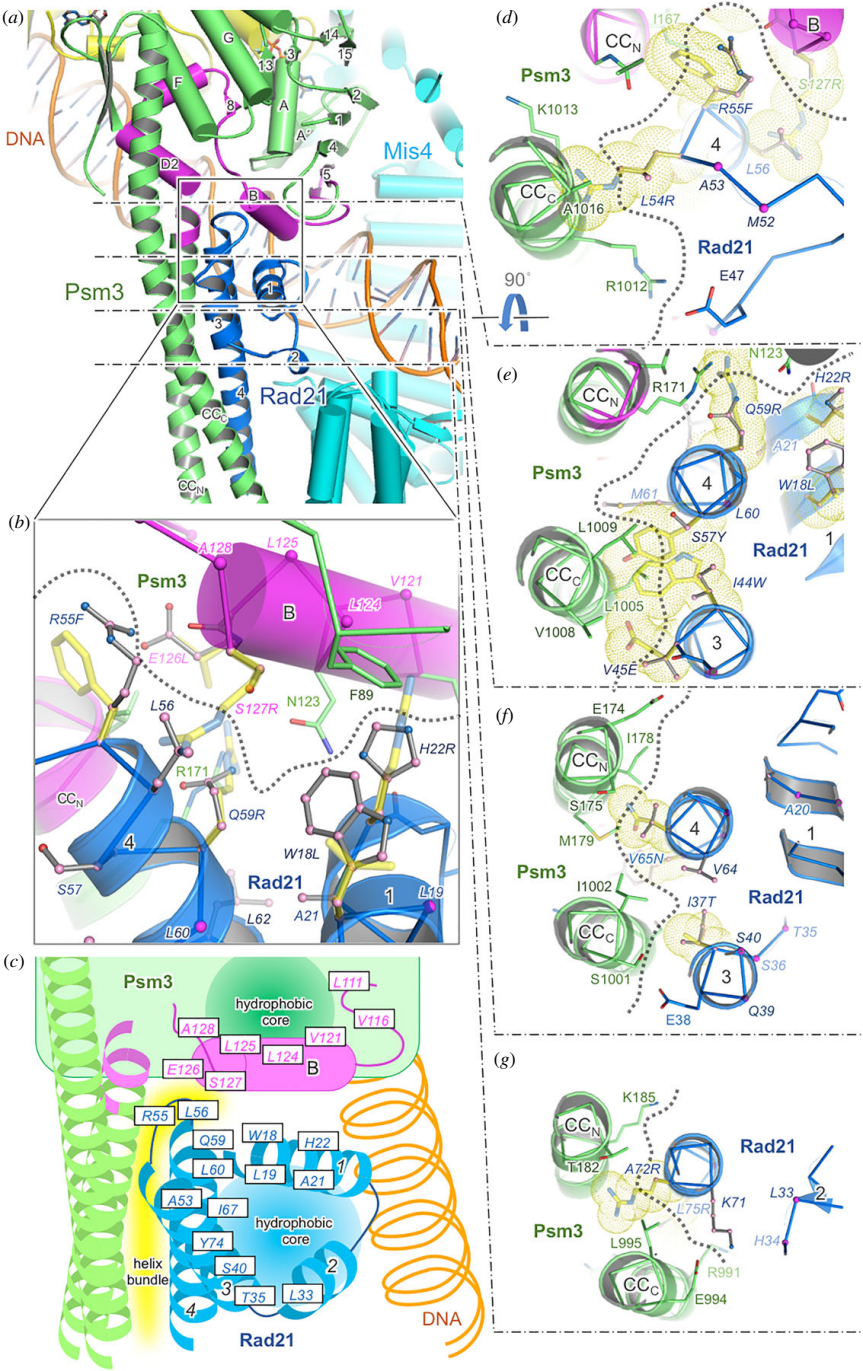
Location of suppressing residues in Psm3-HCJ and Rad21-NTD. (*a*) Subunit interactions between Psm3 and Rad21-NTD in the cohesin complex. (*b*) A detailed view of the subunit interface between the Psm3 head and Rad21-NTD. (*c*) Summarized locations of the suppressor sites identified in subunit interactions between the Psm3 head and Rad21-NTD, and hydrophobic cores. Interaction between Rad21-NTD and Psm3-HCJ is shown with yellow-coloured shapes. (*d–g*) Detailed views of the interface between the Psm3 coiled coil and Rad21-NTD. Yellow, green, blue and cyan are used for Psm1, Psm3, Rad21 and Mis4, respectively, and Psm3-HCJ is coloured in magenta. Suppressor sites are labelled in italics. In panels (*b*) and (*d–g*), suppressor sites located in the subunit interface are represented in a grey ball-and-stick model. C*α* atoms of suppressor sites not located in the subunit interface are represented with magenta spheres. Several suppressing mutations potentially destabilizing the Psm3–Rad21 interaction were manually modelled using the program *Coot* [36] and are represented as yellow transparent sticks and additionally as dotted spheres in panels (*d–g*). The boundary between Psm3 and Rad21 is shown with grey dotted lines.

Rad21-NTD contains four α-helices (α1, α2, α3 and α4) and forms a helix bundle with Psm3 coiled coils close to the Psm3 head domain [19–21]. α3 and α4 form a HTH motif and interact strongly with Psm3 coiled coils (figures 1*a,b*, 2*c,d* and 3). Evolutionary conservation scores for each amino acid along Rad21-NTD were calculated from a multiple sequence alignment of homologous protein sequences (figure 2*c*). Amino acids in the α-helices are more conserved. The turn between helices α3 and α4 has low conservation scores. The 188 single amino acid substitutions in Rad21-NTD that were identified as suppressors of the *cut1-A1816T* ts mutant are shown in a data matrix in figure 2*d*. Single amino acid substitutions in Rad21-NTD are enriched in helices that are evolutionarily conserved (figure 2*c,d*).

### 2.4. Many suppressors are situated on the interface between Psm3 and Rad21

The tertiary structures of the cohesin complex [19,20,37,38] allow us to identify locations of suppressor sites in the cohesin complex and to predict how suppressor mutations affect cohesin's structure (figure 3 and electronic supplementary material, figure S2). The cryo-EM structures show that Rad21-NTD broadly contacts the head and coiled coil of Psm3. In addition to several hydrophilic interactions, extensive hydrophobic interactions form a tight helix bundle between the coiled coil of Psm3 and Rad21-NTD (figure 3*c–e*). In addition, Rad21-NTD is close to the head of Psm3 for potential association. In fact, Arg55 in helix 4 of Rad21-NTD contacts helix B of the Psm3 head in the cryo-EM structures of the cohesin complex with bound DNA and Mis4 [19,37,38].

According to our suppressor screening results, many suppressor mutations occur at residues contributing to Psm3–Rad21 interactions (figure 3 and electronic supplementary material, figure S2). Among amino acids potentially forming hydrophilic subunit interactions, Glu126 in helix B of Psm3 and Arg55 in helix 4 of Rad21-NTD were identified as suppressor sites. These residues are located close to each other to potentially form a salt bridge, which may be disrupted by their replacements with hydrophobic amino acids (figures 2*b,d* and 3*b* and electronic supplementary material, figure S2). Gln59 of Rad21 is located close to the coiled coil and the head of Psm3, and possibly forms a hydrogen bond to it. Its suppressor mutation (Q59R) would disrupt such close contacts between Rad21-NTD and Psm3, because its side chain is much bulkier than that of glutamine. In addition to disruption of these polar contacts, van der Waals contacts between Psm3 and Rad21 should also be disrupted by the suppressors (figure 3 and electronic supplementary material, figure S2). As in several examples shown in figure 3 and electronic supplementary material, figure S2, suppressor sites that have van der Waals contacts with Rad21 or Psm3 were replaced with large hydrophilic amino acids like arginine, bulkier hydrophobic amino acids or smaller amino acids like glycine or proline (figure 2*b,d*).

In addition, many suppressor sites (Leu19, Ala21, Leu33, Thr35, Ser40, Ala53, Leu60, Ile67, Tyr74) were identified in a hydrophobic core that is formed by four helices (α1–4) of Rad21-NTD (electronic supplementary material, figure S2). Such modulation of the hydrophobic core by suppressor mutations is also predicted in the head of Psm3. Many suppressor sites (Leu111, Val116, Val121, Leu124 and Leu125) of Psm3-HCJ were identified on or near helix B of Psm3 (electronic supplementary material, figure S2). One side of helix B in Psm3 forms an intramolecular hydrophobic core and the other side is close to the Rad21-NTD (figure 3*a* and electronic supplementary material, figure S2A). The replacements of the suppressor sites with hydrophilic amino acids, bulkier hydrophobic amino acids or glycine or proline (figure 2*b*) are predicted to change the structure of the hydrophobic cores and potentially cause significant structural re-arrangement of cohesin.

### 2.5. Amino acid preferences were observed

To determine whether there are preferences in wild-type amino acids such that some amino acids occur as mutations more frequently than others, and whether there are preferences in mutant amino acids such that some amino acids are enriched, we calculated the numbers of each wild-type amino acid and the numbers of each mutant amino acid involved in the single amino acid substitutions in Psm3-HCJ and Rad21-NTD (electronic supplementary material, figure S3). Similar patterns were observed for *cut1-A1816T* suppressors in Psm3-HCJ (electronic supplementary material, figure S3A) and in Rad21-NTD (electronic supplementary material, figure S3B). The hydrophobic amino acid leucine (L) in the wild-type amino acid sequence is frequently mutated into other amino acids, while the hydrophilic amino acid arginine (R) and the smallest amino acid glycine (G) are enriched among mutant alleles. In general, hydrophobic interaction is a major driving force for protein folding and is also important in protein stabilization and protein–protein interactions. For these purposes, leucine is often used, as well as other hydrophobic amino acids. In fact, many leucine residues (L111, L124 and L125 in Psm3, and L19, L33, L54, L56, L60, L62, L75 and L76 in Rad21) identified as suppressors of the *cut1-A1816T* ts mutant are involved in the Psm3–Rad21 interaction or the intramolecular hydrophobic core near the subunit interface. Substitution of hydrophobic amino acids, including leucine, with arginine or glycine would cause a structural change around the mutation site by changing the physical volume and/or the electrostatic environment.

### 2.6. The interaction between Psm3-HCJ and Rad21-NTD may be destabilized by suppressors

**Figure 4.**
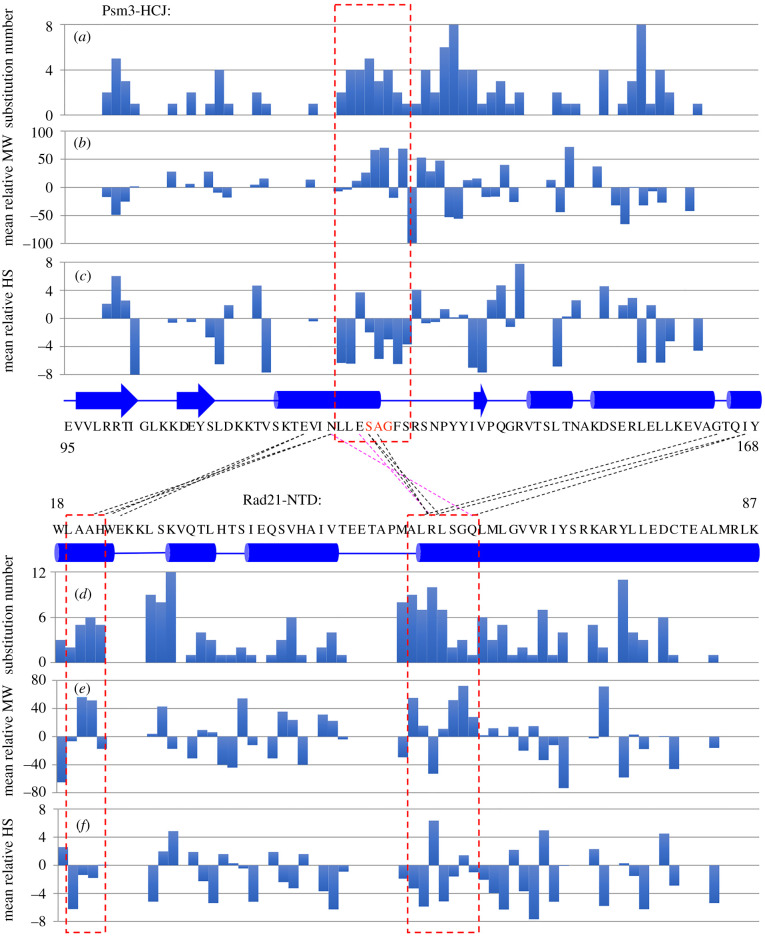
*cut1-A1816T* suppressors tend to impair the interaction between Psm3-HCJ and Rad21-NTD. (*a,d*) Substitution number (the number of amino acids identified as suppressors) at each amino acid position in Psm3-HCJ (*a*) and Rad21-NTD (*d*) is calculated by counting the number of single amino acid substitutions at that residue. (*b,e*) Molecular weights (MWs) of mutant residues in Psm3-HCJ (*b*) and Rad21-NTD (*e*) were compared with those of wild-type residues. Mean relative MWs were calculated for each amino acid. (*c,f*) Hydrophobicity scales (HSs) of mutant residues in Psm3-HCJ (*c*) and Rad21-NTD (*f*) were compared with those of wild-type residues. The mean relative HS was calculated for each amino acid. Black and magenta dashed lines indicate potential van der Waals contacts and hydrogen bonds between residues in Psm3-HCJ and Rad21-NTD predicted according to the cryo-EM structure of the cohesin complex (PDB code: 6YUF). The three red dashed rectangles mark the amino acids that are located at the hydrophobic cores, and single amino acid substitutions in these regions tend to replace wild-type amino acids with larger, more hydrophilic amino acids. Psm3-S127, -A128 and -G129, selected for further analysis, are highlighted in red.

Besides the well-known interaction between Rad21-NTD and Psm3's coiled coil close to its head, Rad21-NTD may also interact with the Psm3 head (figure 4). We realized that Psm3-HCJ amino acid positions 124–131 and Rad21-NTD amino acids 19–22 and 53–59, which are close to each other (figure 3*b* and electronic supplementary material, figure S4), are hot spots that are frequently mutated to other amino acids in *cut1-A1816T* suppressors (figure 4*a,d*). To show the correlation between locations of suppressor sites and changes of physical volume and hydrophobicity of mutated residues more clearly, the mean relative molecular weight (MW) and mean relative hydrophobicity scale (HS) of single amino acid substitutions were calculated at each position in both Psm3-HCJ (figure 4*b,c*) and Rad21-NTD (figure 4*e,f*). Briefly, a positive value of the mean relative MW indicates that the wild-type amino acid at a given position tends to be replaced by larger amino acids and negative values indicate that the wild-type amino acid tends to be replaced by smaller amino acids. Similarly, a positive value of the mean relative HS indicates that amino acid substitutions at that position result in greater hydrophobicity, while a negative value indicates reduced hydrophobicity. The mean relative MWs of amino acids involved in the interaction between Psm3-HCJ and Rad21-NTD are mostly positive, while the mean relative HSs are mostly negative. The results indicate that the single amino acid substitutions at positions located near the close contacts between Psm3 head and Rad21-NTD tend to be bulkier and more hydrophilic, thereby possibly changing the structures of the Psm3 head and Rad21-NTD.

### 2.7. Chromosome segregation defects in *cut1-A1816T* were partially rescued

**Figure 5.**
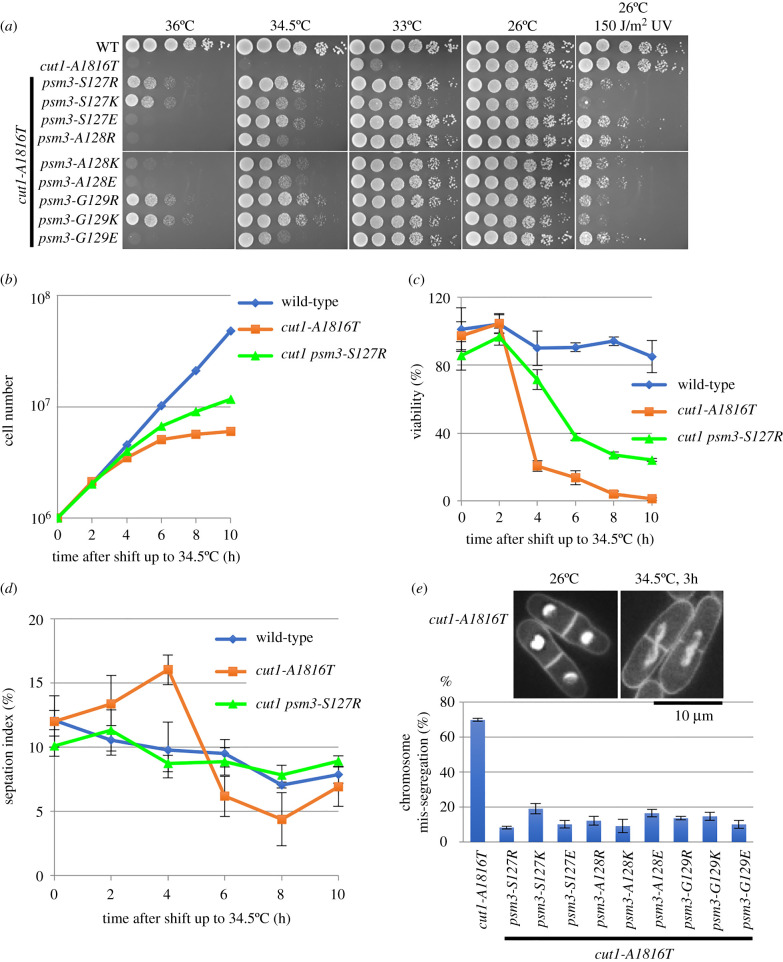
Properties of *cut1-A1816T* suppression by *psm3* mutations. (*a*) Spot test results for *cut1-A1816T* suppressors at the Psm3-S127, -A128 and -G129 positions, which may destabilize the interactions between the Psm3 head and Rad21-NTD. *cut1-A1816T* suppressors rescued its temperature sensitivity, but caused sensitivity to 150 J/m^2^ ultraviolet radiation (UV). (*b*) Cell growth after shifting the temperature of yeast extract–peptone–dextrose (YPD) liquid cultures up to 34.5°C. (*c*) Cell viability after the shifting temperature of YPD liquid cultures up to 34.5°C. Two hundred cells were plated on YPD plates for each condition; the numbers of colonies were counted after incubation at 26°C for 4 days. (*d*) Septation index after shifting the temperature of YPD liquid cultures up to 34.5°C. (*e*) Phenotypes observed under fluorescent microscopy after 4′,6-diamidino-2-phenylindole (DAPI) staining and frequency of chromosome mis-segregation events measured by counting approximately 200 dividing cells after DAPI staining for each strain after incubation at 36°C for 3 h.

Then, we took a closer look at suppression of the *cut1-A1816T* ts mutant by single amino acid substitutions at the Psm3-S127, -A128 and -G129 positions, since they are located at the potential close contacts between the Psm3 head and Rad21-NTD (electronic supplementary material, figure S2) and their substitutions to hydrophilic amino acids (R, K or E) were identified as suppressors of *cut1-A1816T* (figure 2*b*). Spot test results indicated that all these single amino acid substitutions at Psm3-S127, -A128 and -G129 partially rescued the temperature sensitivity of the *cut1-A1816T* ts mutant at the restrictive temperature, but caused sensitivity to DNA damage (figure 5*a*). Further analysis using *cut1-A1816T psm3* double mutants found that suppressors partially rescued the growth defect (figure 5*b*), cell lethality (figure 5*c*), mitotic arrest (figure 5*d*) and chromosome mis-segregation phenotypes (figure 5*e*) observed in the *cut1-A1816T* ts mutant at a restrictive temperature (34.5°C) in liquid culture.

### 2.8. Chromosome segregation in cohesin single mutants

**Figure 6.**
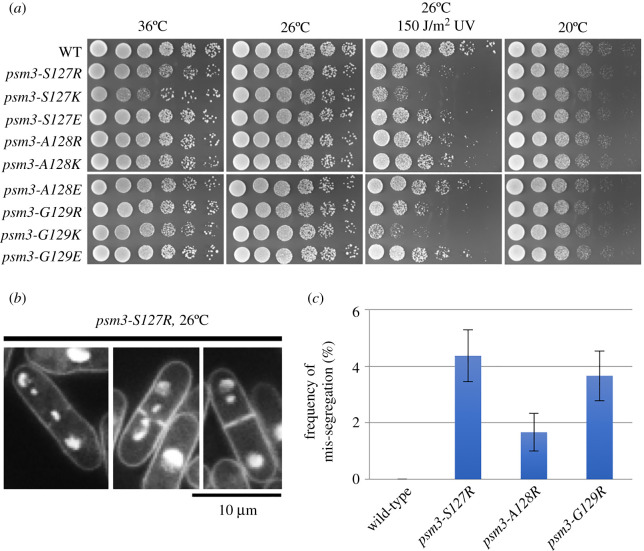
Phenotypes of cohesin single mutants. (*a*) Spot test results of cohesin single mutants at the Psm3-S127, -A128 and -G129 positions. (*b*) Chromosome mis-segregation phenotypes (more than two chromatin dots) were found in the *psm3-S127R* single mutant at 26°C, but at low frequency. (*c*) Quantitative measurement of the frequency of chromosome mis-segregation events in wild-type, *psm3-S127R*, *psm3-A128R* and *psm3-G129R* single mutant cells at 26°C.

Single mutants of *cut1-A1816T* suppressors at the Psm3-S127, -A128 and -G129 positions were generated. They are neither sensitive to high temperature (ts, 36°C) nor sensitive to low temperature (cs, 20°C). However, they are sensitive to 150 J/m^2^ ultraviolet (UV) light (figure 6*a*). UV sensitivities of *psm3* single mutants are consistent with those of *cut1-A1816T psm3* double mutants (figure 5*a*). We observed *psm3-S127R*, *psm3-A128R* and *psm3-G129R* single mutant cells under fluorescent microscopy after 4′,6-diamidino-2-phenylindole (DAPI) staining. Equal chromosome segregation was observed in most mitotic cells, but chromosome mis-segregation phenotypes (more than two chromatin dots) were found occasionally too (figure 6*b*). Then, we measured the frequency of chromosome mis-segregation events quantitatively (figure 6*c*): 1–5% of mitotic cells observed in *psm3-S127R*, *psm3-A128R* and *psm3-G129R* single mutant cells exhibited the chromosome mis-segregation phenotypes (figure 6*c*), but these were not exhibited in the wild-type strain. The results indicate that in cohesin single mutants, which served as suppressors of *cut1-A1816T*, SC separation occurs most efficiently at the metaphase–anaphase transition, but improper SC separations also happen at low frequency.

## 3. Discussion

Spontaneous suppressor screening followed by next-generation whole-genome sequencing identified many mutations at interfaces among cohesin subunits, which were able to bypass the impaired function of Cut1/separase in cleaving cohesin. Saturation mutagenesis in Rad21-NTD and Psm3-HCJ described in this study seemed on the whole to be unbiased and comprehensive. Suppressors of *cut1-A1816T* in both Psm3-HCJ and Rad21-NTD are enriched in evolutionarily conserved amino acid residues, indicating that the mechanism of the suppression may be conserved among species. Psm3-HCJ and Rad21-NTD interact and hotspots for *cut1-A1816T* suppressors were observed at close contacts between Psm3 head and Rad21-NTD. There, single amino acid substitutions tend to replace wild-type amino acids with amino acids that are larger and more hydrophilic, possibly destabilizing the interactions between the Psm3 head and Rad21-NTD, causing cohesin structural re-arrangement.

**Figure 7.**
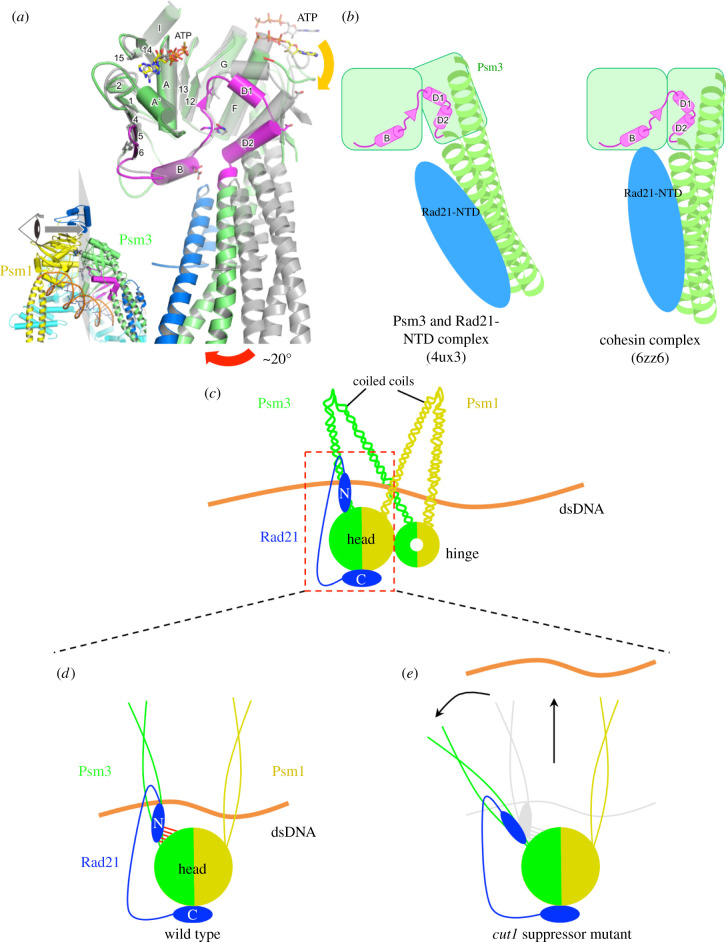
Destabilization of the interactions between Rad21-NTD and the Psm3 head may open closed coiled coils, resulting in release of chromosomal DNA by cohesin. (*a*) A structural difference in the head domain of Psm3 (SMC3) between the cohesin complex with DNA and Mis4(Scc2) bound (PDB code, 6ZZ6), and the Psm3 head complexed with Rad21(Scc1)-NTD (PDB code, 4UX3). Psm3 and Rad21 in the cohesin complex are coloured in green and blue, respectively. The complex of Psm3 with Rad21-NTD, coloured in grey, is aligned at regions 1–149 and 1150–1220 of Psm3. The equivalent region to Psm3-HCJ is coloured in magenta. (*b*) A schematic drawing of the structural difference of the Psm3 head. (*c*) Cohesin SMC dimer is believed to form a folded structure through coiled coils bending around midpoints, which brings the head and hinge domains into proximity. Double-stranded DNA is thought to be clamped by two sets of SMC coiled coils. (*d*) In wild-type cohesin, Rad21-NTD interacts with Psm3 coiled coils close to the head and interacts with the Psm3 head domain (red bars) too. (*e*) In mutant cohesin, single amino acid substitutions may destabilize interactions between Psm3-HCJ and Rad21-NTD, causing Psm3 coiled coils to move away from Psm1 coiled coils and to release the chromosomal DNA.

How can destabilization of the close contacts between the Psm3 head and Rad21-NTD compensate for cohesin-releasing defects in the *cut1-A1816T* ts mutant? Probably they enabled SC separation in a cleavage-independent manner. The importance of the close contacts between hydrophobic cores is reflected by structural comparison of the cohesin complex in the DNA/Mis4(Scc2)-bound state (PDB code, 6ZZ6) [37] with the Psm3(SMC3) complexed with Rad21(Scc1)-NTD (SMC3-HD:Scc1-N complex), in which DNA and Mis4(Scc2) are unbound (PDB code, 4UX3) [20]. The Psm3 coiled coil in DNA/Mis4-bound state rotates approximately 20° inward in comparison with that in the unbound state (figure 7*a,b*) [19,38]. Interestingly, the close contacts between the head of Psm3 and Rad21-NTD only forms in the DNA/Mis4-bound state, and the interaction between the coiled coil of Psm3 and Rad21-NTD persists in both states. It is unclear whether this structural difference depends on DNA/Mis4 binding and/or the heterodimeric formation of Psm3 and Psm1(SMC1), but if it depends on DNA/Mis4 binding the junction between the Psm3 head and coiled coil may serve as a pivot and the interactions between the Psm3 head and Rad21-NTD may contribute to the stabilization of the DNA/Mis4-bound state by maintaining the orientation of the coiled coil of Psm3/SMC3 in close conformation. According to the cohesin ‘ring’ model [39,40], the head and hinge domains are separated by long coiled coils to form a ring-shaped structure that embraces chromosomal DNA. Suppressors of the separase/Cut1 ts mutant should disrupt subunit interactions to open the cohesin ring and to release DNA. However, suppressor mutations that destabilize the interaction between Psm3-HCJ and Rad21-NTD may instead turn the coiled coil orientation to a relatively open conformation, which releases DNA easily.

The cohesin complex is supposed to fold around the midpoints of its coiled coils to bring the head and hinge into proximity [19,30,31,33,37,38,41–44]. Chromosomal DNA may be clamped by the two sets of SMC coiled coils (figure 7*c*) [31,33]. This model explains why suppressor mutations, which are supposed to be distant from the separase/Cut1 cleavage sites in Rad21 (R179 and R231) in the three-dimensional structure of the cohesin complex, can rescue cohesin cleavage defects in separase/Cut1 ts mutants and the lethality of uncleavable Rad21 (*rad21-RERE*, a mutant in which the two separase/Cut1 recognition sites are mutated to glutamic acid and mildly overproduced by plasmid pREP81) [31]. In the wild-type, chromosomal DNA is held tightly by cohesin until its cleavage by activated Cut1/separase. The interactions between the Psm3 head and Rad21-NTD contribute to cohesin's ability to clamp chromosomal DNA (figure 7*d*). Suppressor mutations of the *cut1-A1816T* ts mutant destabilize the interaction between the Psm3 head and Rad21-NTD, which in turn cause cohesin structural re-arrangement that may pivot the Psm3 coiled coils away from the Psm1 coiled coils (figure 7*e*). This may cause loss of the SMC coiled coils' ability to clamp chromosomal DNA tightly and then chromosomal DNA is released from the mutant cohesin without cleavage of Rad21/kleisin by Cut1/separase.

These results thus explain the observed phenotypes. In the wild-type, activated Cut1/separase cleaves Rad21/kleisin to release chromosomal DNA during anaphase (electronic supplementary material, figure S5A). In the *cut1-A1816T* ts mutant at its restrictive temperature, the Cut1/separase function is impaired so that cohesin cannot be cleaved and DNA is not released. Therefore, chromosomes cannot segregate properly and cells die (electronic supplementary material, figure S5B). In *cut1-A1816T* revertants, single amino acid substitutions in Psm3-HCJ or Rad21-NTD destabilize the interactions between Psm3-HCJ and Rad21-NTD, and weaken the grip of SMC coiled coils on chromosomal DNA. Although the function of the mutant Cut1/separase was not recovered, the single amino acid substitutions in Psm3-HCJ and Rad21-NTD enabled chromosome segregation through structural re-arrangement, thereby preventing the lethality caused by the Cut1-A1816T mutation (electronic supplementary material, figure S5C).

## 4. Material and methods

### 4.1. Strains, media and plates

Strains used in this study are listed in electronic supplementary material, table S1. Yeast extract–peptone–dextrose (YPD) medium and plates (1% yeast extract, 2% hipolypeptone, 2% d-glucose) were used for culturing *Schizosaccharomyces pombe* strains.

### 4.2. Liquid culture and phenotypic observation

For cell growth, viability testing, septation indexing and phenotype observation experiments, cells were expended by culturing in YPD liquid medium at the permissive temperature (26°C) overnight. Log-phase cells, after concentration measurement using a cell counter (Sysmex, CDA-100), were diluted to the proper concentration using YPD liquid medium (1 × 10^6^ cells ml^−1^ for cell growth, viability testing and septation indexing experiments; 5 × 10^6^ cells ml^−1^ for phenotypic observation experiments). For viability testing experiments, 200 cells were plated on YPD plates and incubated at 26°C for 4 days. For septation indexing experiments, cell images were taken using an all-in-one fluorescence microscope (Keyence, BZ-X710) and 300 cells were randomly picked up to calculate the septation index for each condition. Chromosome mis-segregation phenotypes were observed using the all-in-one fluorescence microscope (Keyence, BZ-X710) after 20% glutaraldehyde solution (Wako) fixation and DAPI staining; approximately 300 mitotic cells were randomly picked up to calculate the frequency of chromosome mis-segregation phenotypes for each condition.

### 4.3. Site-directed saturation mutagenesis in Psm3-HCJ and Rad21-NTD

A pBluescript plasmid harbouring a nourseothricin sulfate (or clonNAT)-resistant antibiotic marker gene was used for construction of targeting vectors for Psm3-HCJ and Rad21-NTD. A vector with the corresponding wild-type open reading frames (ORFs) integrated upstream of the antibiotic marker gene and approximately 500 bp of sequence after the corresponding ORFs integrated downstream of the antibiotic marker gene was constructed for both Psm3 and Rad21. These plasmids were used as polymerase chain reaction (PCR) templates for saturation mutagenesis. Site-directed PCR-based mutagenesis was then performed to introduce random ‘NNN’ codons (encoding one amino acid) into the Psm3 or Rad21 wild-type ORFs to substitute one amino acid in Psm3-HCJ or Rad21-NTD with all the other potential amino acids or stop codons. In total, 74 and 70 such PCR-based mutagenesis reactions were performed in Psm3-HCJ and Rad21-NTD, respectively. Equal amounts of each PCR product in Psm3-HCJ or Rad21-NTD were mixed to generate two mutation libraries. One mutation library contained all potential single amino acid substitutions in Psm3-HCJ and the other contained all potential single amino acid substitutions in Rad21-NTD. The *cut1-A1816T* ts mutant, chosen for a spontaneous suppressor screen in [31], was used as the host strain. Mutation libraries constructed here were then transformed into the *cut1-A1816T* ts mutant separately, streaked onto YPD plates containing clonNAT (Jena Bioscience, final concentration: 200 µg ml^−1^) and incubated at the restrictive temperature (34.5°C) for 5 days. In total, 387 revertants for Psm3-HCJ and 812 revertants for Rad21-NTD, each containing a single amino acid substitution in Psm3-HCJ or Rad21-NTD in addition to the Cut1-A1816T ts mutation, were isolated. The corresponding single amino acid substitutions in the revertants were determined by targeted sequencing of the corresponding DNA sequences.

### 4.4. Evolutionary conservation score

Nineteen and 14 protein sequences, respectively, of Psm3 and Rad21 homologues in other species were downloaded from the NCBI HomoloGene Database (https://www.ncbi.nlm.nih.gov/homologene) [45]. Protein sequences were aligned using MAFFT, a multiple sequence alignment program (https://mafft.cbrc.jp/alignment/software/) [46]. An evolutionary conservation score for each amino acid residue along the sequence of Psm3 and Rad21 was calculated using protein residue conservation prediction software with the Shannon entropy scoring method [34].

### 4.5. Calculation of mean relative molecular weight and mean relative hydrophobicity scale

A relative MW for each single amino acid substitution in Psm3-HCJ and Rad21-NTD, which was identified as a suppressor mutation of the *cut1-A1816T* ts mutant, was calculated using the formula:

$$\begin{array}{c} \mathrm{Relative}\;\mathrm{molecular}\;\mathrm{weight}=\mathrm{molecular}\;\mathrm{weight}\;o\;\\ \mathrm{mutant}\;\mathrm{allele}-\mathrm{molecular}\;\mathrm{weight}\;\mathrm{of}\;\mathrm{wild}\text{-}\mathrm{type}\;\mathrm{allele}. \end{array}$$

The mean relative MW at every amino acid position in Psm3-HCJ and Rad21-NTD was then calculated as the sum of the relative MWs of every single amino acid substitution identified at that position divided by the number of single amino acid substitutions at that position.

The mean relative HS at each position in Psm3-HCJ and Rad21-NTD was calculated in a similar way. Kyte–Doolittle hydropathy scores of the 20 amino acids [47] were used for the calculation. The relative HS of every single amino acid substitution, identified as a suppressor mutation of the *cut1-A1816T* ts mutant, was calculated using the formula

$$\begin{array}{c} \mathrm{Relative}\;\mathrm{hydrophobicity}\;\mathrm{scale}\;=\mathrm{hydrophobicity}\;\\ \;\mathrm{scale}\;\mathrm{of}\;\mathrm{mutant}\;\mathrm{allele}\;-\;\mathrm{hydrophobicity}\;\mathrm{scale}\;\mathrm{of}\;\\ \;\mathrm{wild}\text{-}\mathrm{type}\;\mathrm{allele}.\; \end{array}$$

The mean relative HS at every amino acid position in Psm3-HCJ and Rad21-NTD was then calculated as the sum of the relative HSs of every single amino acid substitution identified at that position divided by the number of substitutions at the position.

## Data Availability

This article has no additional data.
